# The Yardley Swindle

**Published:** 1872-04

**Authors:** 


### THE YARDLEY SWINDLE.

The following ingenious letter has been sent to all the newspapers in this part of the state, and has been extensively printed, giving the author advertising, free, that would have cost an honest man thousands of dollars:—

"I wish to tell how I cured my cancer last summer without pain or money. Eight years ago a cancer came on my Nose. It grew slow for several years; the last two years it grew very fast. It became frightful. It had begun to eat out my left eye. I had paid hundreds of dollars, and had tried doctors far and near without finding relief. Last summer I drank Wild Tea, putting the tea grounds on my cancer every night as a poultice. In six weeks my cancer was cured. I am now 62 years old. I have given this remedy to several that had cancer, and know many that have been cured since. I believe Wild Tea grows over the country generally, always on high land.

CHARLES YARDLEY,
Pittsburgh, Pa."

Having the same sort of cancer upon our left nose, which nearly destroyed our eye, in the same manner that Mr. Yardley was so sadly afflicted, we wrote to the benevolent old chap—just 62 years old—inquiring more particularly about the "wild tea,"—where it could be plucked, etc., as none of our mountain friends, not even those residing upon high places, had any knowledge whatever, of the yarb.—And here is the reply we received:

"Mr. Charles Yardley, of Pittsburgh, says he is receiving many letters daily in relation to Wild Tea, it is impossible for him to answer so many, he has therefore placed yours in my hands. Yours, Respt.,

J. HENRY SMITH."

Now, what struck us as being very peculiar about the letter of J. Henry Smith is, that his chirography is identical with that of the aforementioned Charles Yardley. Accompanying J. Henry's letter was several printed circulars, extolling the wonderful medical properties of "wild tea," and informing us that for the extremely moderate sum of ten dollars an entire box, containing just one and complete pound of this mountain herb, which grows only upon *high* places, would be sent us, for the cure of the afore mentioned cancer upon our left nose. As yet, we havn't ordered it, but if some infernal fool desires to bite at Mr. Yardley, *alias* J. Henry Smith, *alias* J. B. William's, beautifully engineered little plot for extorting money from the unwary, they can write him to his address below, to wit: J. HENRY SMITH, 362 Liberty St. Pittsburgh, Pa.

